# Reducing healthcare-associated infections incidence by a probiotic-based sanitation system: A multicentre, prospective, intervention study

**DOI:** 10.1371/journal.pone.0199616

**Published:** 2018-07-12

**Authors:** Elisabetta Caselli, Silvio Brusaferro, Maddalena Coccagna, Luca Arnoldo, Filippo Berloco, Paola Antonioli, Rosanna Tarricone, Gabriele Pelissero, Silvano Nola, Vincenza La Fauci, Alessandro Conte, Lorenzo Tognon, Giovanni Villone, Nelso Trua, Sante Mazzacane, for the SAN-ICA Study Group

**Affiliations:** 1 Section of Microbiology and Medical Genetics, Department of Medical Sciences, University of Ferrara, Ferrara, Italy; 2 CIAS Interdepartmental Research Centre, Departments of Medical Sciences and Architecture, University of Ferrara, Ferrara, Italy; 3 Department of Medicine, University of Udine, Udine, Italy; 4 Fondazione Policlinico Universitario Agostino Gemelli, Roma, Italy; 5 Department of Infection Prevention Control and Risk Management, S. Anna University Hospital, Ferrara, Italy; 6 Centre for Research on Health and Social Care Management (CERGAS), Department of Policy Analysis and Public Management, Bocconi University, Milan, Italy; 7 Department of Public Health, Experimental and Forensic Medicine, University of Pavia, Pavia, Italy; 8 Azienda USL di Ferrara, Ferrara, Italy; 9 Department of Biomedical Sciences and Dental and Functional Images, University of Messina, Messina, Italy; 10 Ospedale di Santa Maria del Prato, Feltre (Belluno), Italy; 11 Azienda Ospedaliera Universitaria Ospedali Riuniti di Foggia, Foggia, Italy; 12 Ospedale Sant’Antonio Abate, Tolmezzo (Udine), Italy; University of Calgary, CANADA

## Abstract

Healthcare Associated Infections (HAI) are a global concern, further threatened by the increasing drug resistance of HAI-associated pathogens. On the other hand, persistent contamination of hospital surfaces contributes to HAI transmission, and it is not efficiently controlled by conventional cleaning, which does not prevent recontamination, has a high environmental impact and can favour selection of drug-resistant microbial strains. In the search for effective approaches, an eco-sustainable probiotic-based cleaning system (Probiotic Cleaning Hygiene System, PCHS) was recently shown to stably abate surface pathogens, without selecting antibiotic-resistant species. The aim of this study was to determine whether PCHS application could impact on HAI incidence. A multicentre, pre-post interventional study was performed for 18 months in the Internal Medicine wards of six Italian public hospitals (January 1^st^ 2016—June 30^th^ 2017). The intervention consisted of the substitution of conventional sanitation with PCHS, maintaining unaltered any other procedure influencing HAI control. HAI incidence in the pre and post-intervention period was the main outcome measure. Surface bioburden was also analyzed in parallel. Globally, 11,842 patients and 24,875 environmental samples were surveyed. PCHS was associated with a significant decrease of HAI cumulative incidence from a global 4.8% (284 patients with HAI over 5,930 total patients) to 2.3% (128 patients with HAI over 5,531 total patients) (OR = 0.44, CI 95% 0.35–0.54) (P<0.0001). Concurrently, PCHS was associated with a stable decrease of surface pathogens, compared to conventional sanitation (mean decrease 83%, range 70–96.3%), accompanied by a concurrent up to 2 Log drop of surface microbiota drug-resistance genes (*P*<0.0001; *P*_*c*_ = 0.008). Our study provides findings which support the impact of a sanitation procedure on HAI incidence, showing that the use of a probiotic-based environmental intervention can be associated with a significant decrease of the risk to contract a HAI during hospitalization. Once confirmed in larger experiences and other target populations, this eco-sustainable approach might be considered as a part of infection control and prevention (IPC) strategies.

**Trial registration**—ISRCTN International Clinical Trials Registry, ISRCTN58986947.

## Introduction

Healthcare-associated infections (HAIs) are a global concern impairing the clinical outcome of up to 15% of all hospitalized patients in the world [1]. In Europe, about 3.2 million patients acquire a HAI every year, and 37,000 die as a HAI direct consequence and also because of the increasing multi-drug resistance (MDR) of HAI-associated pathogens [1, 2]. Studies conducted in Italian hospitals show a 5–10% HAI incidence with a mortality rate up to 20–30% [3–5].

On the other hand, it is known that hospital surfaces are persistently contaminated by many microorganisms which can contribute to HAI transmission [6–11], as surfaces represent the reservoir of several pathogens spread by hospital inpatients and personnel [6, 8, 10–15]. Control of surface contamination has been so far approached by conventional chemical-based sanitation, which has limitations, as it cannot prevent recontamination phenomena [16–19], has an high environmental impact, and can contribute to selection of disinfectant-resistant and even antibiotic-resistant pathogens [20, 21], potentially contributing to a further increase of HAI-associated MDR pathogens [22, 23].

Recently, the ‘health’ of hospital surfaces has been re-thought as the health of the human body, considering that, rather than eradicating all pathogens, replacing them by beneficial microbes might be more effective in preventing infections [24, 25]. Toward this principle, a sanitation approach based on eco-sustainable detergents containing spores of *Bacillus* probiotics (Probiotic Cleaning Hygiene System, PCHS) was recently studied, showing that it is safe for hospitalized patients [26], it can stably decrease surface pathogens up to 90% more than conventional disinfectants [27, 28], and it does not select for resistant strains, rather reducing them [29], without increasing sanitation costs [27, 30].

Here we aimed to analyze the impact on HAI incidence by implementing an 18-month multicentre interventional study (from January 1^st^ 2016 to June 30^th^ 2017) in six Italian hospitals, to assess whether the unique use of an innovative eco-sustainable microbial-based cleaning procedure can influence HAI occurrence.

## Methods

### Study design and participants

A multicentre, prospective, pre-post interventional study simultaneously analyzing surface contamination and HAI incidence was conducted in six public medium to large Italian hospitals for 18 months (from January 1^st^ 2016 to June 30^th^ 2017). The trial protocol was approved by the Institutional Ethics Committees of each enrolled healthcare structure. The trial was registered in the ISRCTN Registry (ISRCTN58986947).

Eligibility criteria for enrolled hospitals included: i) approval of local ethical committee before entering the study, ii) presence of internal medicine/geriatrics and neurology wards (which were included in the study), iii) size larger than 100 in-patients beds, iv) presence of an established HAI surveillance program and infection control team, v) acceptance not to introduce any new intervention focused on Infection Control and Prevention (ICP), potentially affecting HAI incidence, except those already existing in the enrolled hospital wards and those necessary to manage possible outbreaks, throughout the whole study.

Enrolled hospitals represented different geographical Italian regions (North, Centre and South), and were randomly allocated in one of two Intervention groups (I_1_, I_2_). One further hospital, meeting all eligibility criteria, was included as an external contemporaneous control (_ext_C), as it did not receive intervention and was only monitored for HAI incidence and environmental bioburden. I_1_-group included three hospitals entering the study on January 1^st^ 2016 (Feltre, Roma, Foggia); I_2_-group included two hospitals entering 5-months later, on May 1^st^ 2016 (Vigevano and Tolmezzo); the _ext_C hospital was monitored starting from May 1^st^ 2016, as for I_2_ group (Messina). Random allocation was performed by an independent investigator using computer-generated random numbers.

The intervention consisted uniquely of the introduction of PCHS sanitation (a patented system by Copma, Ferrara, Italy), replacing the conventional chemical-based (chlorine products) one. Enrolled hospitals agreed not to introduce any other intervention potentially affecting HAI incidence throughout the whole study, except those already existing at the enrolment time. Cleaning staff did not change during the study and were adequately trained for the appropriate PCHS application in all the hospitals receiving the intervention. More precisely, no general cleaning topics were covered, as the training was limited to the correct modalities to prepare and use the PCHS cleansers. No other differences were introduced, either in number and qualification of cleaning staff, nor in frequency of the procedures, resulting in no different perception by cleaning staff, healthcare personnel and patients about the change of the cleaning system. Healthcare personnel, data extractors and patients were not aware about the change of the cleaning system.

The study included two phases: a 6 month pre-intervention period (pre-PCHS), when hospitals maintained the conventional chemical-based sanitizing procedures, and a 6 month post-intervention period (PCHS), when PCHS was routinely applied, with a minimum 2 month interval between the two phases, when PCHS was introduced.

All new patients admitted at the enrolled wards in the pre-PCHS and PCHS periods were included in the study and surveyed for the development of HAIs, without distinction of age or gender and keeping their identity completely anonymous, so that informed consent was not needed. Surveillance of HAIs was already done in all enrolled hospitals, and not implemented for the study, but during the study HAIs were monitored daily, *in continuum*, in order to obtain their true incidence value. Patients already present at the beginning of pre-PCHS and PCHS periods, and in the window period between pre-PCHS and PCHS phases, were excluded. Observation of patients was stopped on the last day of pre-PCHS and PCHS periods.

### HAI analyses

Each HAI occurring during the observation periods in the patients admitted to the enrolled hospital wards in the two observed periods was identified according to the criteria defined by the European Centre for Disease Prevention and Control (ECDC) [31]. All HAI types were included in the study, namely: urinary tract infections (UTI), bloodstream infections (BSI) including those central-vascular catheter (CVC)-related, systemic-clinical sepsis, gastrointestinal infections (GI), skin and soft tissue infections, pneumonia, lower respiratory tract infections (LRI), surgical site infections (SSI), reproductive tract infections, EENT (eye, ear, nose and throat or mouth) infections, bone and joint infections, intra-abdominal infections, and non-specified infections.

HAI etiological agents were identified by microbiology laboratories of each hospital, based on routine diagnostic tests. No changes were applied to the conventional diagnostic process of each hospital, except for *Bacillus* species for which routine searches were done in all clinical samples.

### Environmental sampling and analyses

Hospital surface microbiota was analyzed monthly by a central team (CIAS centre, University of Ferrara). To this aim, three points/room (floor, bed footboard and sink) were sampled in duplicate as previously described [27, 29], in 3–6 randomized rooms/hospital (respectively in hospitals with less or more than 100 enrolled ward beds). Total bacteria, *Staphylococcus* spp., *Enterobacteriaceae* spp., *Acinetobacter* spp., *Mycetes*, *Pseudomonas* spp., and *Clostridium difficile* were quantified on specific Rodac contact plates (CFU/m^2^).

Quarterly (twice in the pre-PCHS and twice in the PCHS phases), the same points were also analyzed by molecular assays, as previously described [29]. Briefly, total bacterial amount, *Bacillus* count and microbiota resistome were respectively quantified by *panB* real time quantitative PCR (*panB*-qPCR), *spo0A*-qPCR and a qPCR microarray for 84 resistance genes (Qiagen Antibiotic Resistance Genes, BAID-1901ZRA, Hilden, Germany). Resistome was also analyzed in four PCHS-*Bacillus* isolates from each sampling campaign of the PCHS-phase.

### Data collection and management

Dedicated healthcare professionals (recruited and trained in a standard way) collected *in continuum* data from patients’ clinical records in a standardised spreadsheet, per each hospital. Professionals collecting clinical data were only aware of an incidence study to be conducted during all the study period (18 months), and were blinded to the intervention time and hospital’s groups.

A first electronic clinical record was filled out for each admitted patient, and contained general data: gender, age, origin, admission date, admission diagnosis, presence of specific risk factors, antibiotic therapy in the two weeks preceding admission, presence of colonization by alert microorganisms, eventual presence of infection at admission and its etiological agent.

A second form, filled out in case of HAI onset, included information about HAI onset, location, etiological agent, drug therapy and infection resolution/outcome. All data were anonymized and submitted centrally via a secure, password-protected website. A central team was available during the whole study period to solve informatics problems, standardizing and validating completeness of data and their consistency. Data analyzers were blinded to the intervention time and hospital’s allocation. A quota corresponding to at least 10 recorded HAIs per hospital setting were validated by a blinded second expert, to minimize the risk of infection miscoding.

Bioburden data, collected monthly by the central team (University of Ferrara), were also uploaded in the same password-protected website.

### Outcome measures

The primary outcome measure was the reduction of HAI incidence in the PCHS compared to the pre-PCHS phase. Variations in infection rates were analyzed both as cumulative incidence per 100 admitted patients, and as HAIs incidence rates per 1,000 patient days. Secondary outcome measures were qualitative and quantitative characterisation of hospital surface bioburden in the surveyed areas.

### Statistical methods

The study power was estimated based on admissions and HAI incidence rates in Italian hospitals [3–5]. The sample size was calculated considering an 80% power to detect an infection incidence reduction of at least 25% starting from a hypothesized rate of 4%, assuming a two-sided test with an alpha level of 0.05, and corresponded to 10,476 patients.

Statistical analyses were performed using chi-square test, Kolmogorov-Smirnov test for evaluating normality, parametric (Student’s *t* test) and non-parametric (Mann-Whitney) tests, chi-square test of association, and multivariable analysis (logistic regression), assuming as statistically significant a *P* value at least <0.05. Multivariable model was developed including all the parameters which showed a statistically significant correlation with HAI occurrence by univariate analysis. Bonferroni correction for multiple comparisons was applied for analysis of microarray data (a *P*_*c*_ value <0.05 was considered significant). Analyses were performed using the software IBM® SPSS20® Statistics (IBM, Bologna, Italy).

### Recruitment

**Fig 1:**
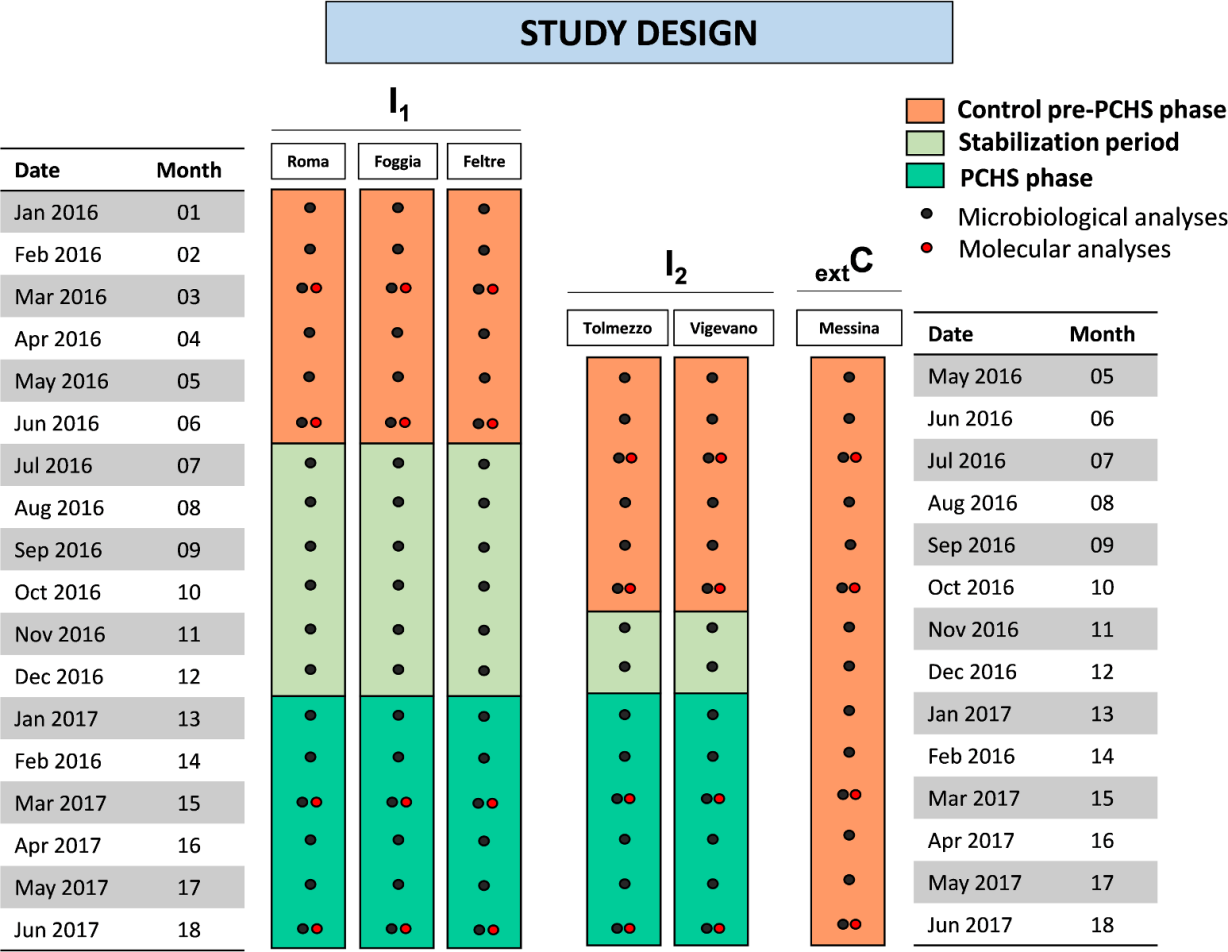
Study design graphic representation. Six Italian hospitals from different geographical regions were enrolled in the study (North: Feltre, Tolmezzo, Vigevano; Centre: Rome; South: Foggia, Messina). Five hospitals were randomly allocated in two Intervention groups (I_1_, I_2_) and one further hospital represented an external control (_ext_C): I_1_-group included Roma, Foggia and Feltre hospitals, entering the study on January 1^st^ 2016; I_2_-group included Vigevano and Tolmezzo hospitals, entering 5-months later, on May 1^st^ 2016; _ext_C hospital was represented by Messina hospital, receiving no intervention and monitored from May 1^st^ 2016. The phases of the study are indicated by colours: orange, 6-months pre-intervention period (pre-PCHS); light green, stabilization period, when PCHS was introduced; green, 6-months post-intervention period (PCHS), when PCHS was routinely applied. Sampling campaigns for microbiological analyses are indicated by circles: conventional microbiological analyses were performed monthly (black circles), and molecular analyses were performed quarterly (red circles) in all enrolled hospitals.

All enrolled hospitals completed the study, guaranteeing a continuous monitoring for a 6-months period in the pre-intervention (pre-PCHS) phase and a 6-months period in the intervention (PCHS) phase (Fig 1). Overall, the study surveyed 11,842 patients, 11,461 from intervention I_1_-I_2_ hospitals and 381 from the external control hospital (Table 1). Globally 24,875 environmental samples were analyzed.

**Table 1:** Population characteristics of study participants in pre-PCHS and PCHS phases, stratified by enrolled hospitals.

		Patients No.			Age (mean±SD)		Length of stay (mean±SD)		Patients with at least one HAI No. (%)		
Group	Healthcare Structure	Total	Pre-PCHS	PCHS	Pre-PCHS	PCHS	Pre-PCHS	PCHS	Pre-PCHS	PCHS	Statistical significance
**I**_**1**_	Feltre	2,812	1,599	1,213	73.1±16.4	74.9±15.4	8.7±5.7	10.0±6.1	77 (4.8%)	30 (2.5%)	*P* = 0.0013 OR, 0.50 95% CI, 0.33–0.77
	Foggia	1,951	966	985	72.4±15.9	74.7±14.8	9.9±5.4	12.0±7.1	106 (11.0%)	36 (3.7%)	*P*<0.0001 OR, 0.31 95% CI, 0.21–0.45
	Roma	3,116	1,611	1,505	68.0±17.8	68.1±17.2	10.4±8.9	11.0±7.3	50 (3.1%)	20 (1.3%)	*P* = 0.0008 OR, 0.42 95% CI, 0.25–0.71
**I**_**2**_	Tolmezzo	2,453	1,186	1,267	74.3±14.3	75.9±13.3	10.6±9.8	9.8±6.3	25 (2.1%)	21 (1.7%)	*P* = 0.4111 OR, 0.78 95% CI, 0.44–1.41
	Vigevano	1,129	568	561	72.7±15.5	72.6±16.1	8.9±5.4	9.6±6.2	26 (4.6%)	21 (3.7%)	*P =* 0.4829 OR, 0.81 95% CI, 0.45–1.46
**Tot. (I**_**1**_**+I**_**2**_**)**		11,461	5,930	5,531	71.8±16.4	73.0±15.8	9.7±7.6	10.5±6.7	284 (4.8%)	128 (2.3%)	*P*<0.0001 OR, 0.47 95% CI, 0.38–0.58
_**ext**_**C**	Messina	381	146	235	71.3±14.9	72.3±15.7	11.6±8.9	9.7±5.7	12 (8.2%)	16 (6.8%)	*P* = 0.6080 OR, 0.82 95% CI, 0.37–1.78

I_1_, Intervention group 1; I_2_, Intervention group 2; _ext_C, external control hospital.

## Results

### Impact of sanitation on HAI incidence

Globally 11,842 patients were surveyed, all hospitalized in the Internal Medicine wards of the enrolled hospitals, this aimed to analyze the incidence of HAIs in a more homogeneous patients’ sample possible.

Table 1 summarizes the main characteristics of the studied population by a participating hospital. HAI cumulative incidence (patients with HAI/enrolled patients) decreased significantly in the PCHS period compared to the pre-PCHS phase of the I_1_-I_2_ hospitals, from 4.8% (283/5,930) to 2.3% (128/5,531) (range 1.3–3.7%) (*P*<0.0001), regardless of the geographical location and entering time in the study. HAI incidence rate per 1,000 patient-days decreased from 5.4 (314/57,742) to 2.4 (141/58,201), with an incidence rate ratio of 0.45 (95% CI, 0.36–0.54). The decrease was evident in each individual hospital, including the structure with a previous very low HAI incidence (Tolmezzo, from 2.1% to 1.7%). A slight not significant reduction was observed also in the external control hospital, where the total number of HAIs was 15 infections in 12 patients in the first 6-month period, and 16 infections in 16 patients in the second one, with a respective cumulative incidence rate of 8.2% and 6.8% (OR = 0.82; 95% CI, 0.37–1.78; *P* = 0.6), and a relative incidence rate per 1,000 patient-days corresponding to 9.4 (15/1,600) and 7.0 (16/2,279) respectively (OR = 0.75; 95% CI, 0.37–1.54).

**Fig 2:**
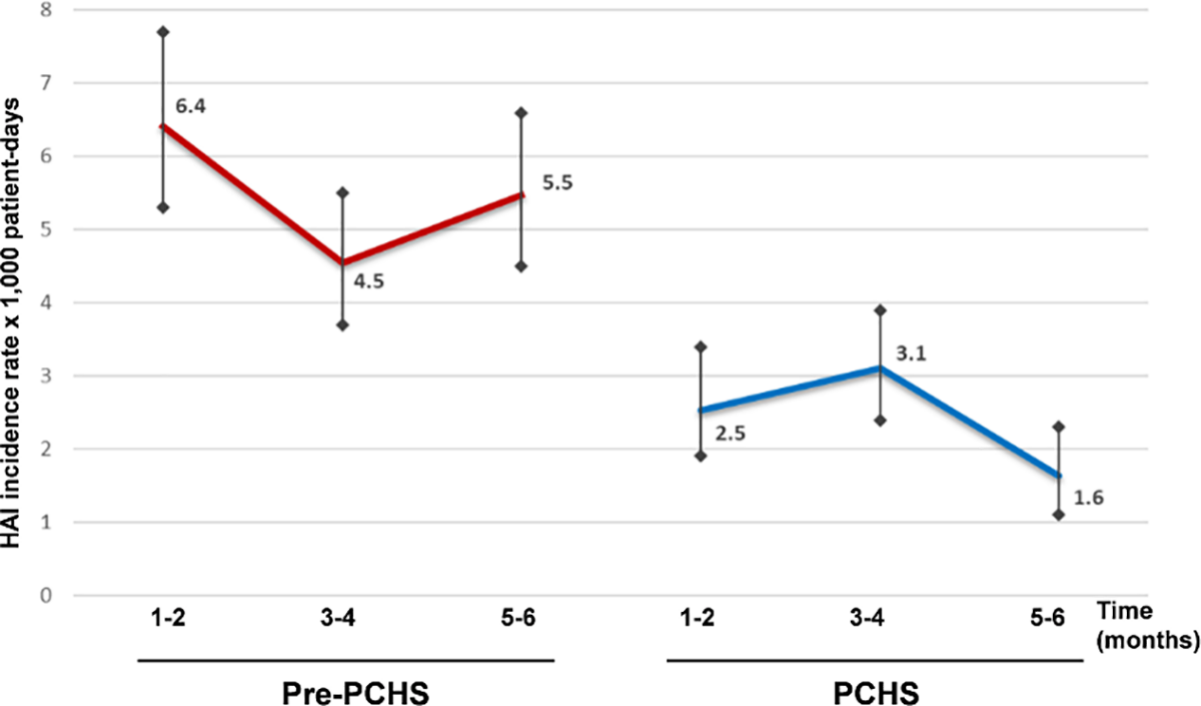
HAI incidence rates in the I_1_-I_2_ intervention hospitals. Results are expressed as bimonthly value of incidence rate per 1,000 patient-days, respectively in the pre-PCHS (red) and PCHS periods (blue). 95% CI intervals are also reported.

The analysis of HAI incidence bimonthly rates in the pre-PCHS and PCHS phases, showed no tendency to decrease in the pre-PCHS period, prior to the intervention, whereas a stable reduction was observed following the introduction of PCHS sanitation (Fig 2).

The main clinical features of the observed patients were very similar in the pre-PCHS and PCHS periods of the study, as shown in Table 2. The univariate analysis results confirmed as risk factors for HAI occurrence those already reported in the literature, indicating for example a positive correlation with the presence of urinary or central venous catheters and increasing age, whereas a protective effect emerged for being a male and self-sufficiency (S1 Table).

**Table 2:** Patient characteristics of the I_1_-I_2_ hospitals in the pre-PCHS and PCHS periods (11,461 patients).

Patients characteristics	Pre-PCHS	PCHS
	Total patients No. (%)	Total patients No. (%)
**Total**	**5,930**	**5,531**
**Gender: male**	2,977 (50.2%)	2,928 (52.9%)
**Age <65**	1,518 (25.6%)	1,265 (22.9%)
**Age 65–74**	1,261 (21.3%)	1,177 (21.3%)
**Age 75–84**	1,821 (30.7%)	1,753 (31.7%)
**Age ≥85**	1,330 (22.4%)	1,336 (24.2%)
**Incontinence**	1,448 (24.4%)	1,369 (24.8%)
**Disorientation**	804 (13.6%)	747 (13.5%)
**Self-sufficiency**	3,671 (61.9%)	3,632 (65.7%)
**Pressure sores**	393 (6.6%)	237 (4.3%)
**Surgery 30 day before**	122 (2.1%)	80 (1.4%)
**Ventilation**	215 (3.6%)	161 (2.9%)
**Parenteral nutrition**	200 (3.4%)	141 (2.5%)
**ATB 2 week before**	566 (9.5%)	294 (5.3%)
**MDRO at admission**	131 (2.2%)	83 (1.5%)
**Infection at admission**	1,216 (20.5%)	1,089 (19.7%)
**Urinary catheter (any type)**	1,368 (23.1%)	1,166 (21.1%)
**CVC**	264 (4.5%)	260 (4.7%)

Self-sufficiency, ability to provide for themselves autonomously, measured by SSM (Self Sufficiency Matrix) scale; ATB, antibiotics; MDRO, multi drug resistant organism; CVC, central vascular catheter.

Among all the observed HAIs, urinary tract infections (UTI) represented the most prevalent infection type (Table 3), followed by bloodstream infections (BSI), systemic clinical sepsis, gastrointestinal infections (GI), skin and soft tissue infections, and respiratory infections. Following PCHS intervention, the cumulative incidence of the most frequent HAIs decreased: UTI, from 3% (179/5,930) to 1.2% (70/5,531); bloodstream infections-BSI, from 0.9% (54/5,930) to 0.6% (31/5,531); clinical sepsis, from 0.4% (22/5,930) to 0.1% (5/5,531); gastro-intestinal infections from 0.3% (17/5,930) to 0.1% (6/5,531); and skin/soft tissue infections from 0.3% (16/5,930) to 0.1% (6/5,531). Instead, the relative burden of each HAI type did not change significantly in the PCHS phase compared to the pre-PCHS one.

**Table 3:** HAIs in pre-PCHS and PCHS phases, stratified by type.

		Pre-PCHS (I_1_ + I_2_) No. (%)	PCHS (I_1_ + I_2_) No. (%)	_ext_C 1^st^ 6-months period No. (%)	_ext_C 2^nd^ 6-months period No. (%)
**No. of HAIs**		314* (100%)	**141° (100%)**	**15**^**#**^ **(100%)**	**16**^**§**^ **(100%)**
**Type**	Urinary tract infections-UTI	179 (57.0%)	70 (49.6%)	8 (53.3%)	6 (37.5%)
	Bloodstream infections-BSI [CVC related]	54 [10] (17.2%)	31 [7] (22.0%)	-	3 (18.8%)
	Clinical sepsis	22 (7.0%)	5 (3.5%)	1 (6.7%)	-
	Gastrointestinal-GI	17 (5.4%)	6 (4.3%)	2 (13.3%)	-
	Skin and soft tissue	15 (4.8%)	6 (4.3%)	3 (20.0%)	1 (6.2%)
	Pneumonia	12 (3.8%)	8 (5.7%)	-	2 (12.5%)
	Lower respiratory tract	10 (3.2%)	6 (4.3%)		3 (18.8%)
	Reproductive tract	1 (0.3%)	-	-	-
	Eye, ear, nose and throat or mouth EENT	1 (0.3%)	2 (1.4%)	-	-
	Bone and joint	-	1 (0.7%)	-	-
	Intra-abdominal	-	1 (0.7%)	-	-
	Surgical Site Infection	-	-	-	1 (6.2%)
	Not specified	3 (1.0%)	5 (3.5%)	1 (6.7%)	-

* 256 patients with 1 HAI, 26 with 2 HAIs, and 2 with 3 HAIs

° 115 patients with 1 HAI, and 13 with 2 HAIs

^#^ 9 patients with 1 HAI, and 3 with 2 HAIs

^§^ 16 patients with 1 HAI

Similarly, the number of HAI-associated microorganisms (identified in HAI patients) decreased significantly from 332 in the pre-PCHS phase to 137 in the PCHS phase (Table 4), whereas the relative percentages of isolated microorganisms remained unaltered: *E*. *coli*, *E*. *faecalis*, *S*. *aureus*, *P*. *mirabilis* and *P*. *aeruginosa* were in fact the most frequently isolates in both phases. Importantly, no infections sustained by PCHS-derived *Bacilli* were detected in any of the hospitalized patients in the enrolled structures, further supporting the absence of infectious risks associated with PCHS-*Bacilli* use indicated by previous studies.

**Table 4:** Microorganisms isolated from HAIs during pre-PCHS and PCHS phases in I_1_-I_2_ hospitals.

	Pre-PCHS	PCHS	
Infections*	301	135	
**Exam not available or negative**	27	19	
**Exam available**	274	116	
**Isolated microorganisms**	**Samples (n,%)**	**Samples (n,%)**	**PCHS *vs* pre-PCHS**
*S*. *aureus*	21 (6.3%)	16 (11.6%)	-23.8%
*Staphylococcus spp*.	30 (9.0%)	10 (7.2%)	-66.6%
*Enterococcus spp*.	57 (17.2%)	24 (17.5%)	-57.8%
*Streptococcus spp*.	7 (2.1%)	4 (2.9%)	-42.8%
*C*. *difficile*	9 (2.7%)	3 (2.2%)	-66.6%
*E*. *coli*	93 (28%)	27 (19.7%)	-70.9%
*Klebsiella spp*.	19 (5.7%)	12 (8.7%)	-36.8%
*P*. *mirabilis*	15 (4.5%)	6 (4.3%)	-60.0%
*P*. *aeruginosa*	15 (4.5%)	10 (7.2%)	-33.3%
*Enterobacter spp*.	8 (2.4%)	1 (0.7%)	-87.5%
*Citrobacter spp*.	3 (0.9%)	0	-100%
*A*. *baumannii*	8 (2.4%)	5 (3.6%)	-37.5%
*Morganella spp*.	3 (0.9%)	0	-100%
Other *Enterobacteriaceae*	1 (0.3%)	0	-100%
*Candida spp*.	26 (7.8%)	11 (8.0%)	-57.7%
Virus	5 (1.5%)	3 (2.1%)	-40.0%
Others	12 (3.6%)	5 (3.6%)	-58.3%
**Total**	**332 (100%)**	**137 (100%)**	**-**

*During pre-PCHS phase, 301 HAIs included 13 co-infections; during PCHS phase, 135 HAIs included 6 co-infections.

The relative role of PCHS in the reduction of HAI onset was explored by a multivariable model including all the parameters emerged as variables positively associated with HAI occurrence by univariate analysis. The results (summarized in Table 5), while confirming as statistically significant risk factors the presence of urinary or central venous catheters (respectively OR = 2.68; 95% CI, 2.10–3.41 and OR = 1.99; 95% CI 1.40–2.82), showed PCHS use as a statistically significant independent protective effect (OR = 0.44; 95% CI, 0.35–0.54) (*P*<0.0001).

**Table 5:** Risk factors associated with HAI onset in patients of I_1_-I_2_ hospitals: Multivariable model*.

Population characteristics	*P*	OR	95% CI
**Male**	0.01812	0.78	0.63–0.96
**Age 65–74 *vs* Age <65**	0.0047	1.71	1.18–2.48
**Age 75–84 *vs* Age <65**	0.0004	1.88	1.33–2.67
**Age 85 or more *vs* Age <65**	0.0026	1.78	1.22–2.58
**Length of stay**	p<0.0001	1.08	1.07–1.09
**Incontinence**	0.2253	0.85	0.66–1.10
**Disorientation**	0.0226	1.37	1.05–1.76
**Self-sufficiency**	0.5600	0.92	0.69–1.43
**Pressure sores**	0.9757	0.99	0.69–1.44
**Ventilation**	0.7702	1.07	0.68–1.67
**ATB 2 week before**	0.8479	0.97	0.68–1.37
**MDRO at admission**	0.6230	0.86	0.47–1.57
**Urinary catheter (any type)**	p<0.0001	2.68	2.10–3.41
**CVC**	0.0001	1.99	1.40–2.82
**PCHS**	p<0.0001	0.44	0.35–0.54

* multivariable model included all the factors emerged as significantly associated with HAI onset by univariate analysis (11,461 patients).

### Impact of sanitation on hospital surface microbiota

Surface bioburden analyses, including detection and quantification of *Staphylococcus* spp., *Enterobacteriaceae* spp., *Pseudomonas* spp., *Acinetobacter*, *Clostridium difficile* and *Candida* spp., showed a persistent contamination in all the enrolled wards in pre-PCHS period, with an overall pathogen load corresponding to 22,737 CFU/m^2^ (median value, range 17,053–60,632 CFU/m^2^), mostly attributable to Staphylococcal contamination (median load 21,895 CFU/m^2^, range 13,684–57,263 CFU/m^2^). Other microbial genera were less abundant: *Enterobacteriaceae* (median value 1,784 CFU/m^2^; range 444–3,015 CFU/m^2^), *Acinetobacter* (mean value 2,538 CFU/m^2^; range 214–3,836 CFU/m^2^), *Pseudomonas* spp. (mean value 361 CFU/m^2^; range 43–2,125 CFU/m^2^), *C*. *difficile* (mean value 286 CFU/m^2^; range 137–842 CFU/m^2^) and *Candida* spp. (mean value 1,480 CFU/m^2^; range 1,075–5,508 CFU/m^2^) (supporting information files in BioStudies repository, Accession No. S-BSST75).

**Fig 3:**
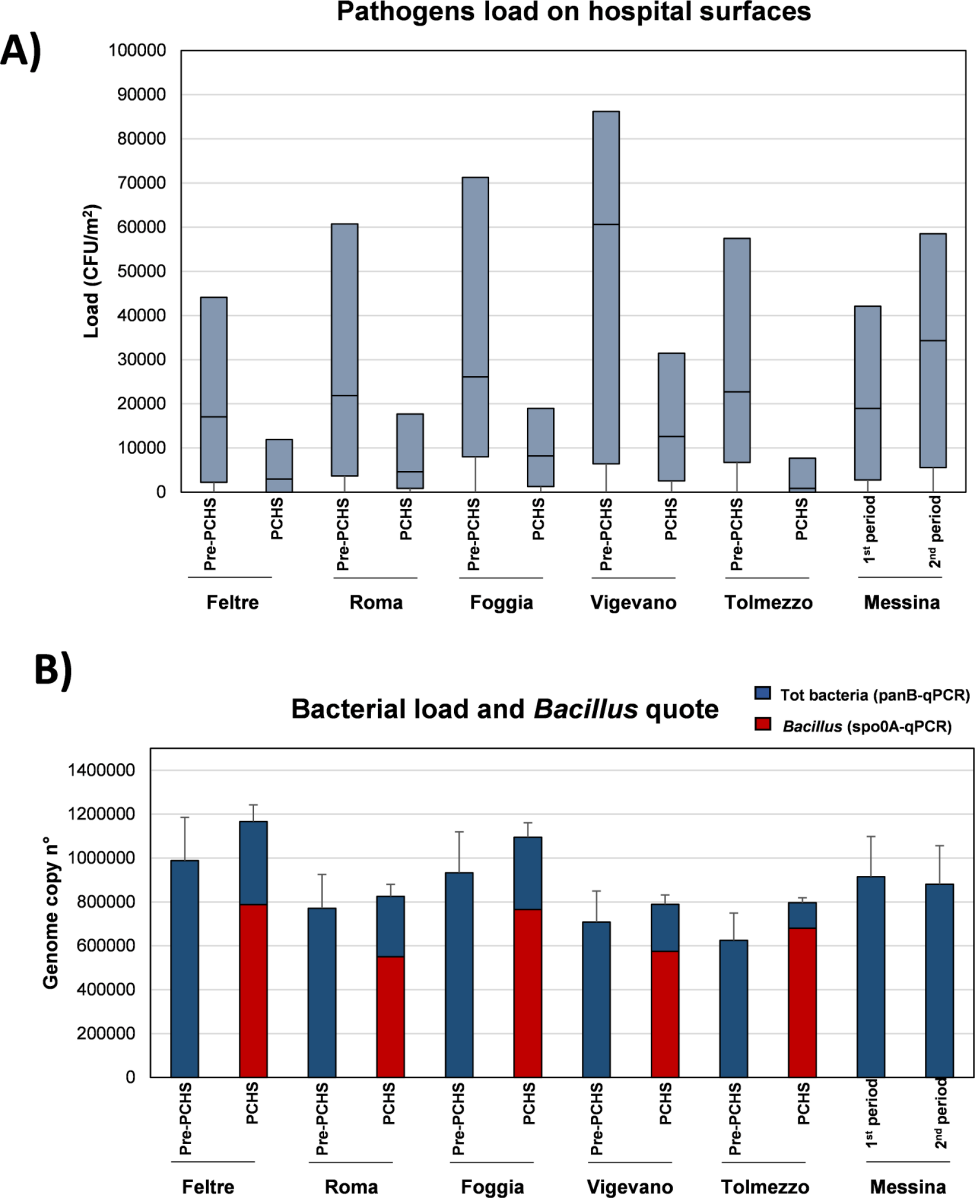
Surface contamination in the surveyed hospitals. (A) Pathogen load on hospital surfaces, expressed as CFU/m^2^. Six pathogens were measured by direct CFU counting on specific Rodac plates, as described in Methods (*Staphylococcal* spp., *Enterobacteriaceae* spp., *Acinetobacter* spp., *Candida* spp., *Pseudomonas* spp., *Clostridium* spp.). Graphed results represent the sum of the median values obtained for each measured pathogen. Median values (lower part of the box) and Q3 values (upper part of the box, representing the 75% percentile values) are shown for each hospital, and for pre-intervention (pre-PCHS) and intervention (PCHS) phases. Values reported for the external control hospital (Messina), correspond to those detected in the 1^st^ and 2^nd^ 6-month periods of the study. (B) Total bacterial load and PCHS-*Bacilli* count, respectively measured by a pan-bacterial qPCR (*panB*) and a specific qPCR for *Bacillus* genus (*spo0A*). Results are expressed as genome copy number per 100 ng of tested DNA. The median values ± SD of pre-PCHS and PCHS phases are shown. Values reported for the external control hospital (Messina), correspond to those detected in the 1^st^ and 2^nd^ 6-month periods of the study.

The introduction of PCHS in the five intervention hospitals (I_1_ and I_2_ groups) induced a statistically significant decrease of pathogen contamination from 22,737 CFU/m^2^ to 4,632 CFU/m^2^ (median value; range 842–12,632 CFU/m^2^) (*P*<0.0001, corresponding to a mean 83% decrease of surface pathogen load (range 70–96.3%) (Fig 3A). By contrast, no variations were observed in the external control hospital between the two observation periods.

Meanwhile, the quota of PCHS-*Bacilli* increased significantly on surfaces of intervention-hospitals from 0% (median value, range 0–30%) to 69.8% (median value, range 39.9–86.8%) of the total surface microbiota (*P*<0.0001) (Fig 3B). No increase in *Bacillus* counts was observed in the _ext_C control hospital.

Microarray analysis of the microbiota resistome showed a significant global decrease of resistance genes in the I_1_-I_2_ hospitals during the PCHS-phase compared to what detected in the pre-PCHS period (*P*<0.0001; *P*_*c*_ = 0.008)(S1 Fig)(supporting information files in BioStudies repository, Accession No. S-BSST75). The prevalence of R genes was different in the individual hospitals, likely reflecting the selective pressure exerted in each setting, but the decrease of the R genes originally present during the pre-PCHS phase was observed in all hospitals. No decrease was instead observed in the external control hospital.

**Fig 4:**
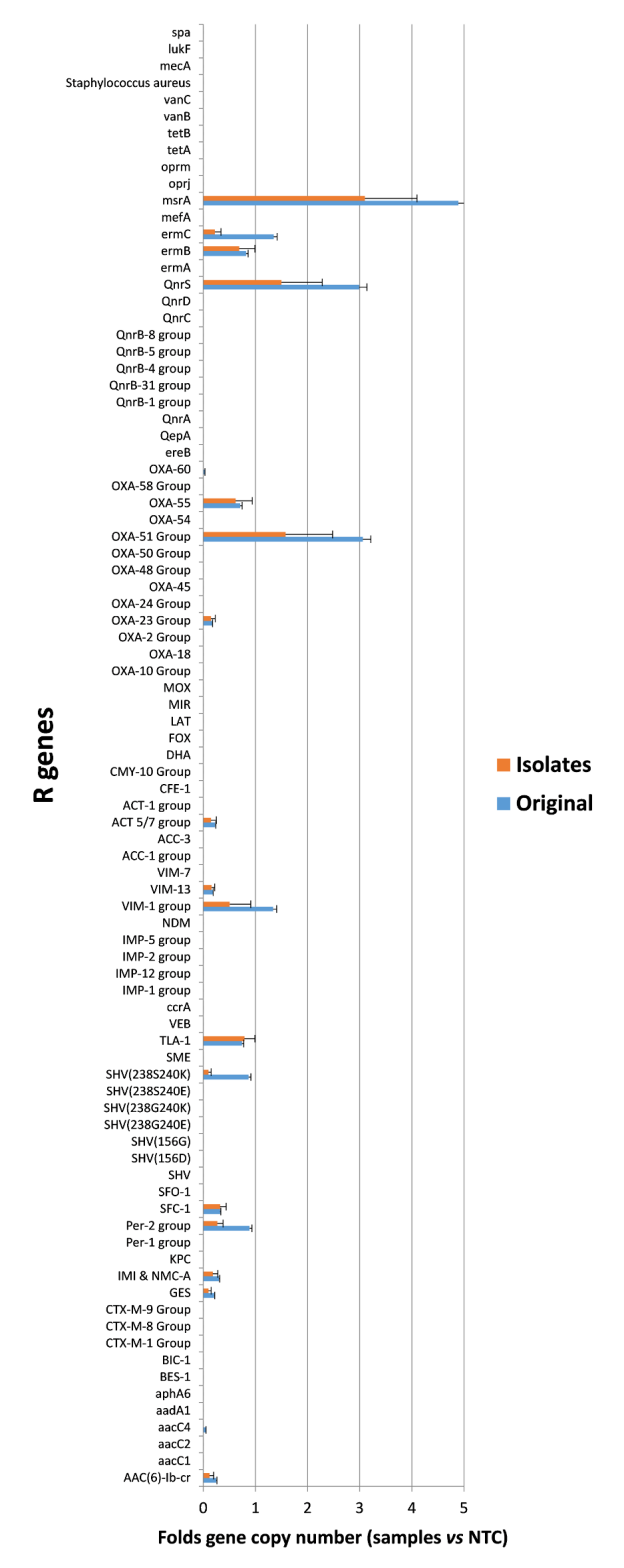
Resistome analysis of PCHS-*Bacillus* strains. Antibiotic resistance genes were analyzed by microarray both in the PCHS detergent prior to application, containing a blend of three *Bacillus* species (Original) and in the *Bacillus* isolates (Isolates) collected from hospital surfaces in the PCHS phase of I_1_ and I_2_ hospital groups. For original PCHS-*Bacilli*, results are expressed as mean values ± SD of six replicates. For Isolates, results are expressed as the mean value ± SD of 120 *Bacillus* isolated from hospital surfaces. Both Original and Isolates values were compared to negative control values (NTC). Each *Bacillus* isolate was identified by PCR and sequencing prior to microarray analysis, as previously described [29].

In parallel, resistome microarray analysis of PCHS-*Bacilli* isolates from surfaces of treated hospitals, showed no acquisition of R genes in all tested isolates during the whole study period (Fig 4), confirming previous studies supporting the genetic stability of the PCHS-*Bacillus* strains.

## Discussion

The role of persistent surface contamination in HAI transmission is recognized [6, 8–11], but the impact of environmental cleaning on HAI incidence lacks of robust data, since so far most studies correlating environmental bioburden with HAIs incidence considered a bundle of factors, or were limited to specific ward types (i.e. ICU)[32, 33].

Since we recently reported that a probiotic-based sanitation (PCHS) can modulate surface hospital microbiota [27, 29], the present pre-post interventional study was aimed to investigate directly the potential impact of this system on HAI incidence.

According to our results, in the absence of any other ICP intervention, PCHS was associated with a significant reduction (*P*<0.0001) of HAI incidence in the medical wards of acute hospitals. The reduction was observed in all HAIs commonly detected in internal medicine wards, some of which are associated with contact transmission, and this was associated with a concurrent decrease of the responsible isolated microorganisms, while their relative frequencies remained unaltered.

Multivariable analysis, while confirming the role of well known risk factors [34], such as the presence of catheters (OR = 2.68 and OR = 1.99 for urinary and CVC, respectively), revealed PCHS to be an independent protective factor (OR = 0.44; 95% CI, 0.35–0.54) (*P*<0.0001).

Furthermore, the bioburden data confirmed in a large sample that PCHS is able to reduce and remodulate the environmental contamination, inducing a significant decrease (-83%) of the overall surface pathogen load as well as of the resistance genes harboured by the surface microbiota (up to 2 Logs) [27–29], suggesting that probiotic *Bacilli* can displace and replace pre-existing pathogens, limiting colonization and spreading of new potentially pathogenic and drug-resistant entries (contamination from care-givers, new patients, healthcare workers), a competitive mechanism well known in nature [35–38].

In addition, microbiological and molecular systematic monitoring of the present study supports the safety of use of PCHS observed in previous trials [26, 27, 39], confirming the genetic stability of the PCHS-*Bacilli* and the absence of any infectious risk correlated to the use of PCHS probiotics in hospital settings.

### Limitations

Although showing a strong protective effect of PCHS, this study has some potential limitations. A first possible one is related to the study design, which is a pre-post intervention run in the same hospitals. Nevertheless, the size of the sample and the magnitude of the resulting reduction seem to indicate a clear role of PCHS. Based on these results, further developments could include studies based on larger samples and different methodologies, such as stepped wedge trials and/or cluster randomized trials, including cost effectiveness. Also, the numbers of the external control hospital are small, and the non significant reduction observed might be due to this limitation. Since the explored settings are limited to internal medicine, geriatrics and neurology, further studies would benefit from exploring the impact in other healthcare settings, in order to better understand the generalizability of the obtained results.

Secondly, the sample size was calculated to detect differences in the global sample and not in the individual hospitals; nevertheless, the results showed a HAI decrease in all hospitals, although it was not statistically significant in those hospitals with a low HAI incidence at baseline.

Thirdly, a potential bias might be represented by the awareness of the healthcare personnel about the study itself, but healthcare professionals were aware only of an incidence study to be conducted during the whole study period (18 months). Furthermore, this point was addressed by limiting the information exclusively to hospital managers, recruiting external data collectors and data extractors, and including an external control hospital to monitor the potential impact related only to the presence of a study. At the same time it should be considered that the period of the study was very long (all together the span of time was 18 months), thus limiting the potential attention bias of the healthcare workers teams.

A further potential bias could be related to seasonality and geographical distribution of the enrolled hospitals. The 6 month follow-up period may not be sufficient to negate the effects of seasonal variability, and similarly the lack of enrolled sites from all three regions in both I_1_ and I_2_ groups may be a limitation. Further studies could therefore include a longer follow-up period and/or a more robust enrolment. Nevertheless, the size and the characteristics of the HAI reduction and the relative effect in term of displacement of microbiota seems to indicate that these potential confounders, if existing, could have a limited effect.

Lastly, although there was an agreement not to introduce measures to improve infection control in the enrolled hospitals, a potential for confounding is represented by the lack of measurement of hand hygiene over the study period.

### Conclusions

This is the first study, to our knowledge, which shows an association between HAI incidence and environmental microbiota in such a large sample. Overall, collected results may contribute to emphasize the role of environmental microbiota modulation for cleaning in healthcare settings, introducing the possibility of an ecological approach in the area of environmental cleaning, which might be included among the effective tools available for infection prevention and control (IPC). This could support policies aimed at reducing the development of microbial resistance to disinfectants and antibiotics, leading to an effective reduction of costs related to HAI management. On another hand, our results might be useful to introduce methodologies to investigate environmental bioburden and circulation of resistome in healthcare settings, as its systematic analysis might open the possibility to explore new strategies in controlling its spread. Last, this study opens new issues to be explored: the applicability and the impact in different settings, the impact on different types of HAIs, the long-term effect of the routine use of PCHS, the dynamics between human pathogens population and probiotic *Bacilli* and the impact on costs related to management of HAIs. Of course, deep analyses about cost effectiveness will be needed, as well as future studies optimally designed to address information still lacking in this study.

In conclusion, these results might be important to better understand the role of environmental microbiota in healthcare settings, supporting the development of guidelines about environmental cleaning addressed to enhance IPC strategies.

## Supporting information

S1 Table HAI frequency in relation to patient characteristics in I_1_-I_2_ hospitals. Univariate analysis results of risk factors for HAI occurrence in Pre-PCHS and PCHS patients.
(DOCX)

S1 Fig Resistome analysis of the surface microbiota. (A) Analysis of the antibiotic resistance genes in the whole bacterial surfaces population of the five hospitals subjected to intervention (I_1_-I_2_ hospitals), in the pre-PCHS and PCHS phases of the study. Results are expressed as mean ± SD fold changes, compared to negative control values (for the pre-PCHS phase) and to pre-PCHS values (for PCHS phase). (B) Analysis of the antibiotic resistance genes in the whole surface microbiota of the external control (_ext_C) hospital, in the 1^st^ and in the 2^nd^ 6-month periods of the study. First-period results are expressed as mean fold changes ± SD compared to negative control values (NTC); 2^nd^-period results are expressed as mean values ± SD compared 1^st^-period values, similarly to what performed for pre-PCHS and PCHS phases in the hospitals subjected to intervention.
(TIF)
