# Supplementary material for: Reducing healthcare-associated infections incidence by a probiotic-based sanitation system: A multicentre, prospective, intervention study
Source: PLoS One. 2018 Jul 12;13(7):e0199616. doi: 10.1371/journal.pone.0199616 (PMC6042698; doi:10.1371/journal.pone.0199616)
Supplement: S1 Table — Univariate analysis results of risk factors for HAI occurrence in Pre-PCHS and PCHS patients. (DOCX) [file pone.0199616.s001.docx]

**S1 Table. HAI frequency in relation to patient characteristics in I_1_-I_2_ hospitals.**

| **Patients characteristics** | **Pre-PCHS** | **PCHS** |  |  |
| --- | --- | --- | --- | --- |
|  | Patients with HAIs  No. (%) | Patients with HAIs  No. (%) | **OR** | **95% CI** |
| **Total** | **284 (4.8%)** | **128 (2.3%)** |  |  |
| **Gender: male** | 119 (4.0%) | 57 (1.9%) | 0.69 | 0.57-0.85 |
| **Age <65** | 38 (2.5%) | 12 (0.9%) | - | - |
| **Age 65-74** | 67 (5.3%) | 25 (2.1%) | 2.14 | 1.51-3.04* |
| **Age 75-84** | 107 (5.9%) | 44 (2.5%) | 2.41 | 1.74-3.33^§^ |
| **Age ≥85** | 72 (5.4%) | 47 (3.5%) | 2.55 | 1.83-3.57° |
| **Incontinence** | 99 (6.8%) | 47 (3.4%) | 1.72 | 1.40-2.11 |
| **Disorientation** | 69 (8.6%) | 31 (4.1%) | 2.12 | 1.68-2.67 |
| **Self-sufficiency** | 122 (3.3%) | 57 (1.6%) | 0.42 | 0.35-0.52 |
| **Pressure sores** | 28 (7.1%) | 13 (5.5%) | 1.96 | 1.41-2.74 |
| **Surgery 30 day before** | 3 (2.5%) | 1 (1.3%) | 0.54 | 0.20-1.45 |
| **Ventilation** | 19 (8.8%) | 9 (5.6%) | 2.24 | 1.51-3.34 |
| **Parenteral nutrition** | 10 (5.0%) | 3 (2.1%) | 1.07 | 0.61-1.87 |
| **ATB 2 week before** | 41 (7.2%) | 5 (1.7%) | 1.58 | 1.15-2.16 |
| **MDRO at admission** | 8 (6.1%) | 6 (7.2%) | 1.91 | 1.10-3.31 |
| **Infection at admission** | 48 (3.9%) | 20 (1.8%) | 0.78 | 0.60-1.01 |
| **Urinary catheter (any type)** | 144 (10.5%) | 64 (5.5%) | 3.82 | 3.14-4.66 |
| **CVC** | 37 (14.0%) | 16 (6.2%) | 3.32 | 2.45-4.49 |

***,** Age 65-74 *vs* Age <65

^§^, Age 75-84 *vs* Age <65

°, Age 85 or more *vs* Age <65

Self-sufficiency, ability to provide for themselves autonomously, measured by SSM (Self Sufficiency Matrix) scale; ATB, antibiotics; MDRO, multi drug resistant organism; CVC, central vascular catheter.
