# Supplementary figures and images for: Reducing healthcare-associated infections incidence by a probiotic-based sanitation system: A multicentre, prospective, intervention study
Source: PLoS One. 2018 Jul 12;13(7):e0199616. doi: 10.1371/journal.pone.0199616 (PMC6042698; doi:10.1371/journal.pone.0199616)

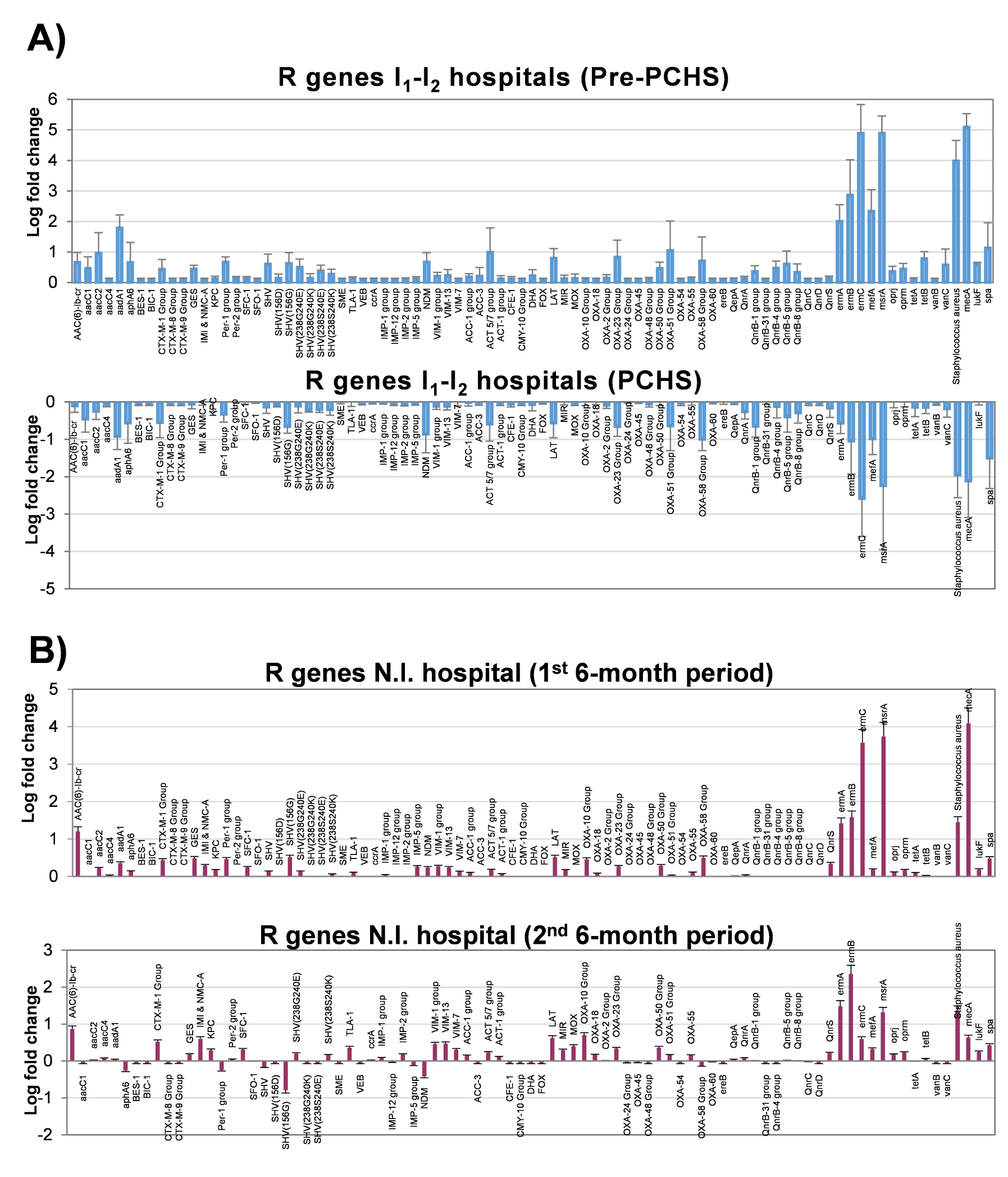

Supplement: S1 Fig — (A) Analysis of the antibiotic resistance genes in the whole bacterial surfaces population of the five hospitals subjected to intervention (I1-I2 hospitals), in the pre-PCHS and PCHS phases of the study. Results are expressed as mean ± SD fold changes, compared to negative control values (for the pre-PCHS phase) and to pre-PCHS values (for PCHS phase). (B) Analysis of the antibiotic resistance genes in the whole surface microbiota of the external control (extC) hospital, in the 1st and in the 2nd 6-month periods of the study. First-period results are expressed as mean fold changes ± SD compared to negative control values (NTC); 2nd-period results are expressed as mean values ± SD compared 1st-period values, similarly to what performed for pre-PCHS and PCHS phases in the hospitals subjected to intervention. (TIF) [file pone.0199616.s002.tif]
